# Supplementary material for: Flexible and Transparent Luminescent Cellulose-Transition Metal Cluster Composites
Source: Nanomaterials (Basel). 2023 Jan 31;13(3):580. doi: 10.3390/nano13030580 (PMC9920715; doi:10.3390/nano13030580)
Supplement: Supplementary file 1 [file nanomaterials-13-00580-s001.zip › nanomaterials-2160423 supplementary.pdf]

nanomaterials

MDPI

Supplementary Materials

Flexible and Transparent Luminescent Cellulose-Transition Metal Cluster Composites

Maria Amela-Cortes *, Noée Dumait, Franck Artzner, Stéphane Cordier and Yann Molard *

Université de Rennes, CNRS, ISCR – UMR 6226, ScanMAT – UAR 2025, IPR – UMR 6251, F-35000 Rennes, France

* Correspondence: maria.amela-cortes@univ-rennes1.fr (M.A.-C.); yann.molard@univ-rennes1.fr (Y.M.)

I. Materials and preparation

Cluster complex $Cs_2Mo_6I_8(OCOCF_2CF_5)_6$ was synthetized starting from $Cs_2Mo_6I_{14}$ and silverpentafluoropropionate following published procedures.[1] Cellulose Acetate Phtalate (CAP) Mw = 2534,12 was purchased from Fluka.

Cluster complex@CAP mixtures and film deposition

Amounts of cluster complex and CAP were calculated to obtain 2 g of composite sample. Two different solutions were prepared. One contained 1, 10, 20 or 30 wt% of cluster complex dissolved in 5 mL of HPLC grade acetone. The second one was obtained by dissolving CAP in HPLC grade acetone by heating at 50 °C for 30 min. Once all CAP was dissolved, the corresponding solution of cluster complex was added and the mixture stirred for 1h at 50 °C. Finally, the mixtures were poured into glass petri dishes and the solvent was slowly evaporated.

II. Experimental techniques

FTIR spectroscopy

CAP, MoIP cluster complex, and composite films were all analyzed by infrared spectroscopy using Universal Attenuated Total Reflectance Accessory FT-IR Spectroscopy (Bruker, vertex 70). Spectra were recorded in the range from 500 to 5 000 cm^{-1} and analyzed using OPUS software.

Thermal analysis

DSC measurements were carried out in a DSC 25 differential scanning calorimeter (TA Instruments, New Castle, DE, USA), calibrated using purified indium (99.9%) as the standard reference material. Samples (3–4 mg) were cut off and placed in an aluminum pan. They were heated at a constant rate of 10 K min^{-1}, using dry atmosphere of argon as carrier gas, in a temperature range of 30 to 200 °C.

Thermogravimetric analyses were performed at 10 K min^{-1} on a TGA/DT Perkin Pyris Diamond.

Small Angle X-ray scattering (SAXS)

X-ray diffraction patterns were collected with a Mar345 Image-Plate detector (Maresearch) mounted on a rotating anode X-ray generator FR591 (Bruker-AXS) operated at 50 kV and 50 mA with Cu Kα radiation (λ = 1.541 Å). The sample to detector distance (422 mm) has been calibrated by using silver behenate. The X-ray patterns were therefore recorded for a range of reciprocal spacing $q = 4\pi\sin\theta/\lambda$ from 0.04-2.5 Å-1 where θ is the diffraction angle. The experiments performed with the present set-up provide accurate measurements of distances between 150 Å and 3 Å. The acquisition time was 1 hour. Film

samples were placed perpendicularly to the beam The scattering intensities as a function of the radial wave vector were determined by circular integration.

Photophysical properties

Transmission measurements were recorded on a Perking Elmer Lambda 35 UV-vis spectrophotometer.

Lifetime measurements and TRPL mapping were realized using a picosecond laser diode (Jobin Yvon deltadiode, 375 nm) and a Hamamatsu C10910-25 streak camera mounted with a slow single sweep unit. Signals were integrated on a 30 nm bandwidth. Fits were obtained using origin software and the goodness of fit judge by the reduced 2 value and residual plot shape. The luminescence spectra in deaerated dichloromethane and absolute quantum yields in the solid state were measured with a C9920–03 Hamamatsu system equipped with a 150 W xenonlamp, a monochromator, an integrating sphere and a red-NIR sensitive PMA-12 detector.

III. Tables and Figures

Table S1. Wavenumbers in cm^{-1} of CAP, MoIP and hybrids MoIP@CAP in film form by ATR-FTIR.

Sample	(C=O)	(o-phtalate)	(C-O-C) / pyranose	(o-phtalate)
**CAP**	1727	1599, 1580	1034	743
**MoIP**	1669			
**MoIP1@CAP**	1674	1597, 1579	1029	743
**MoIP10@CAP**	1670	1597, 1579	1033	745
**MoIP20@CAP**	1680	1585	1039	741
**MoIP30@CAP**	1687	1597,1586	1026	741

a)

c)

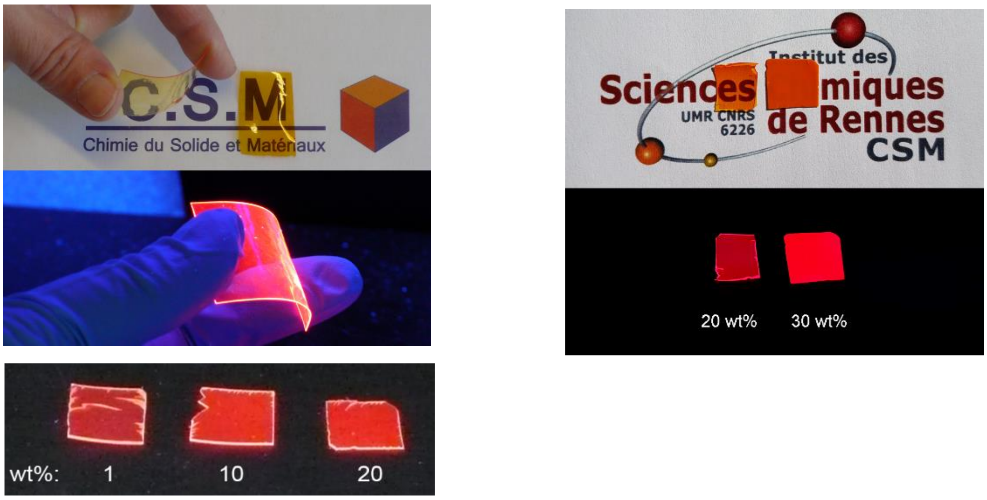

Figure S1. Pictures of the films a) MoIP1@CAP and MoIP10@CAP under day light and MoIP1@CAP under 365 nm light, b) MoIP20@CAP and MoIP30@CAP under day light and under 365 nm and c) CAP doped at 1, 10 and 20 wt% under 365 nm light.

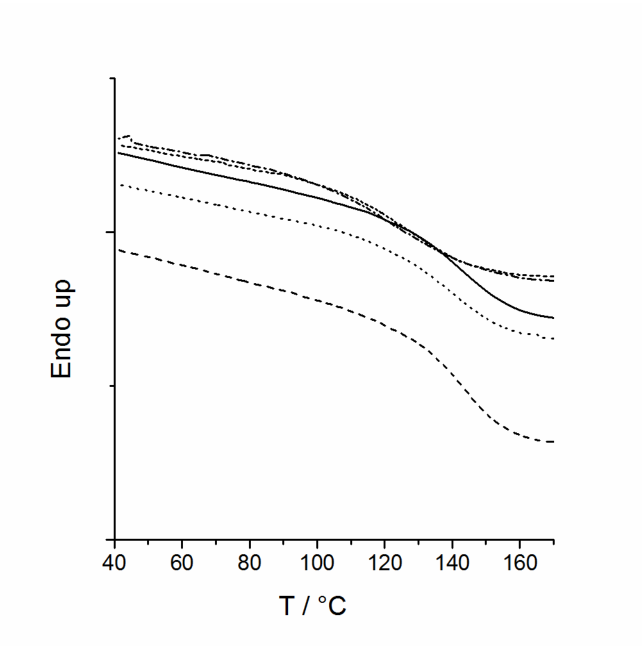

Figure S2. Thermograms of Cellulose films doped with $Cs_2Mo_6I_8(C_2F_5COO)_6$: CAP (straight), MoIP1@CAP (dash), MoIP10@CAP (dot), MoIP20@CAP (dash dot), MoIP30@CAP (short dash) on cooling at 10 $K.min^{-1.}$

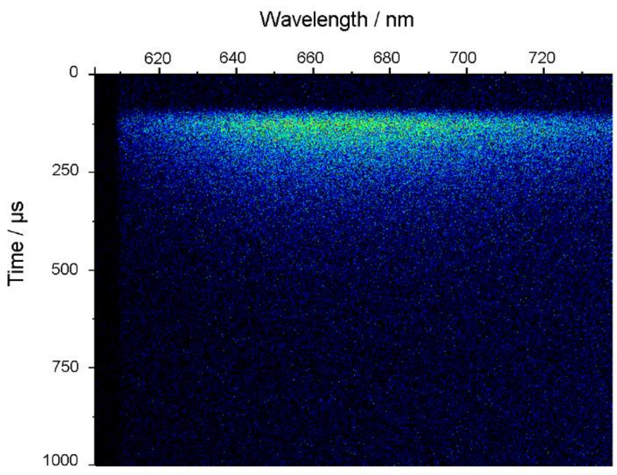

Figure S3. Thin film emission decay map of MoI1@CAP.

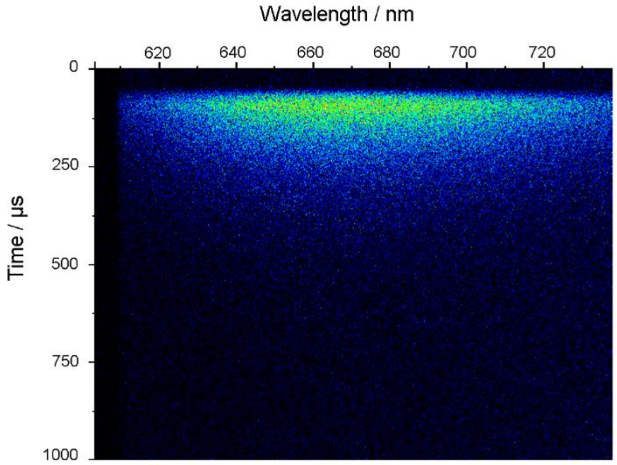

Figure S4. Thin film emission decay map of MoI10@CAP.

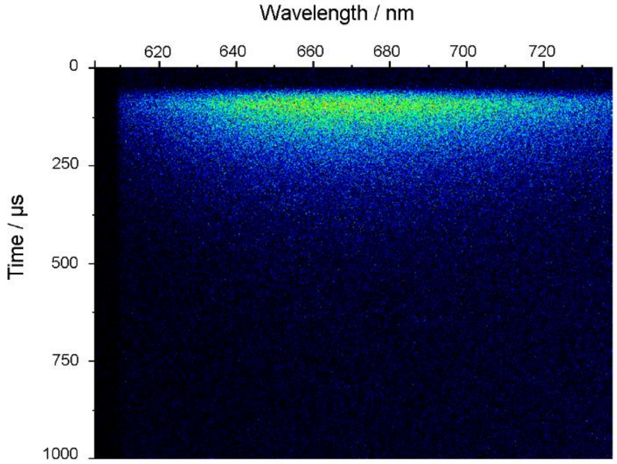

Figure S5. Thin film emission decay map of MoI20@CAP.

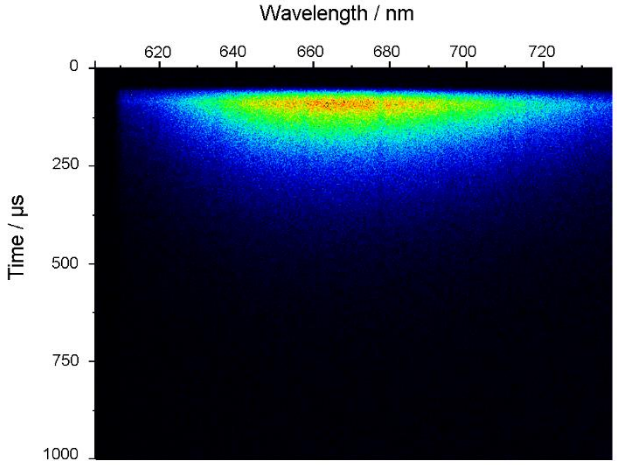

Figure S6. Thin film emission decay map of MoI30@CAP.

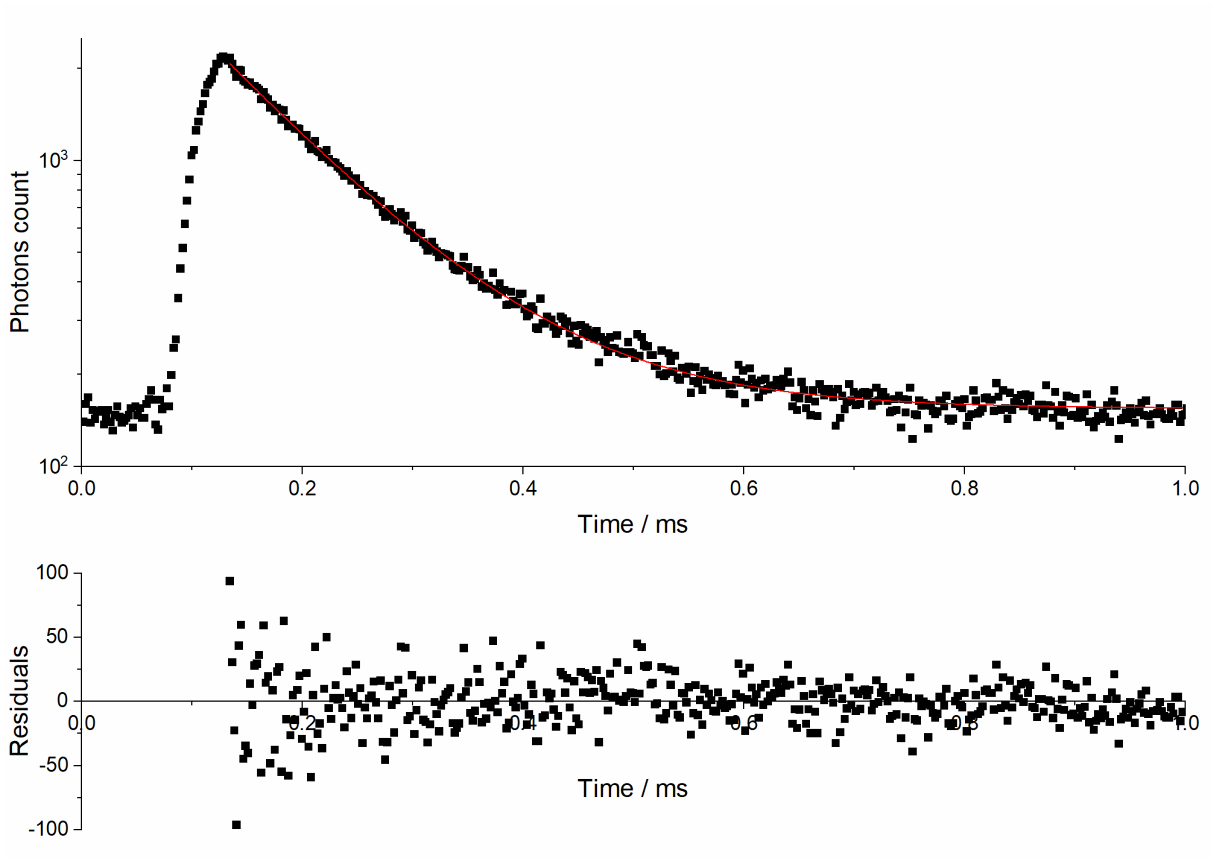

Figure S7. Emission decay profile (black squares, top), corresponding fitted curve (red line) and fit residuals(bottom) for **MoI1@CAP.**

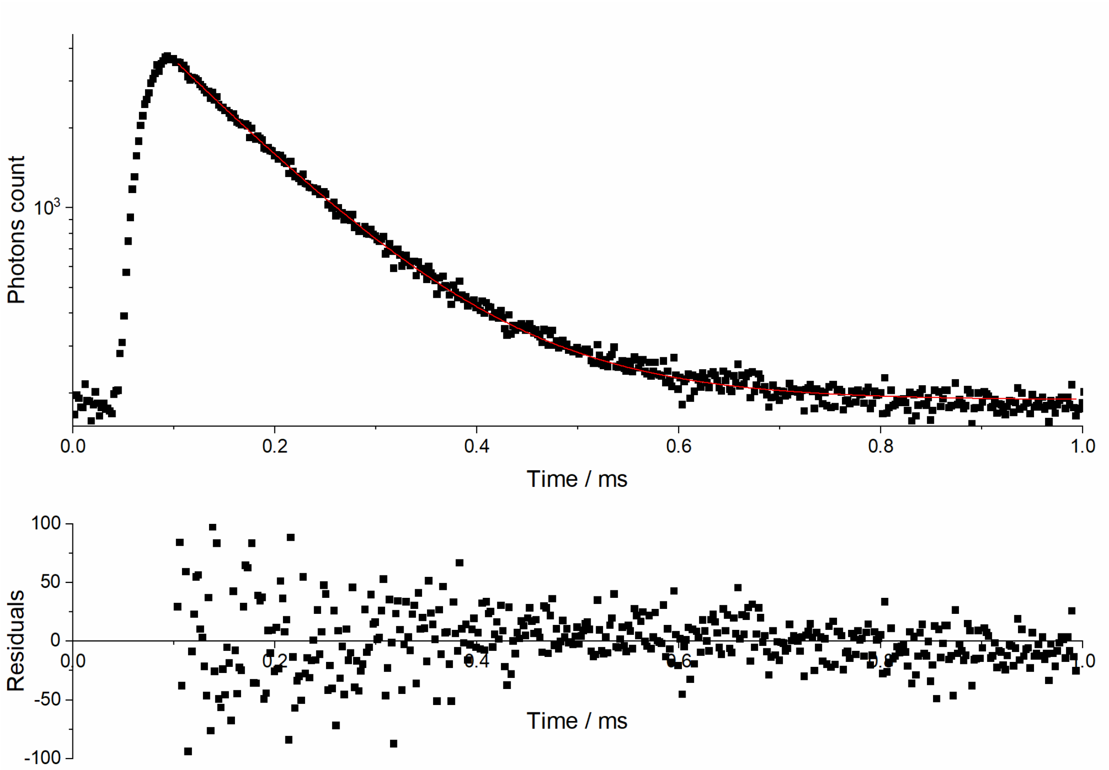

Figure S8. Emission decay profile (black squares, top), corresponding fitted curve (red line) and fit residuals(bottom) for **MoI10@CAP.**

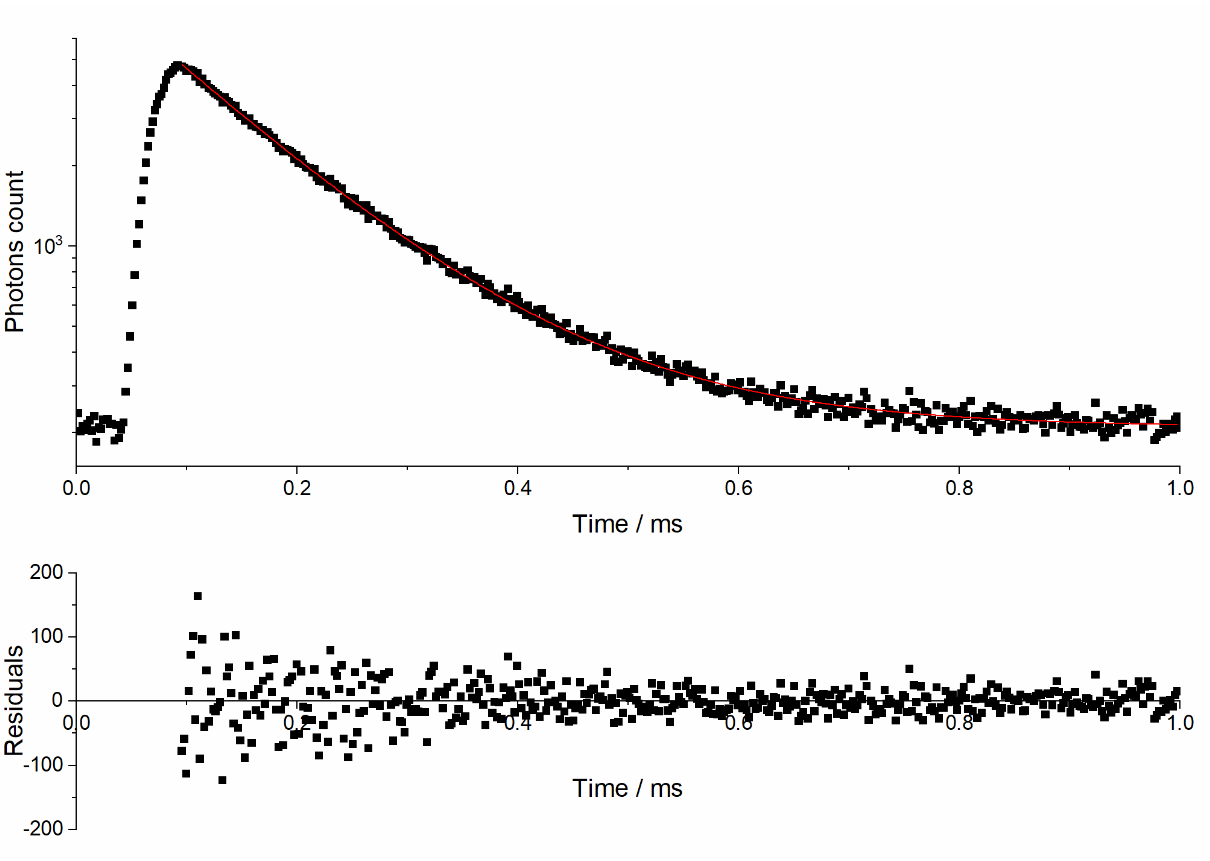

Figure S9. Emission decay profile (black squares, top), corresponding fitted curve (red line) and fit residuals(bottom) for **MoI20@CAP.**

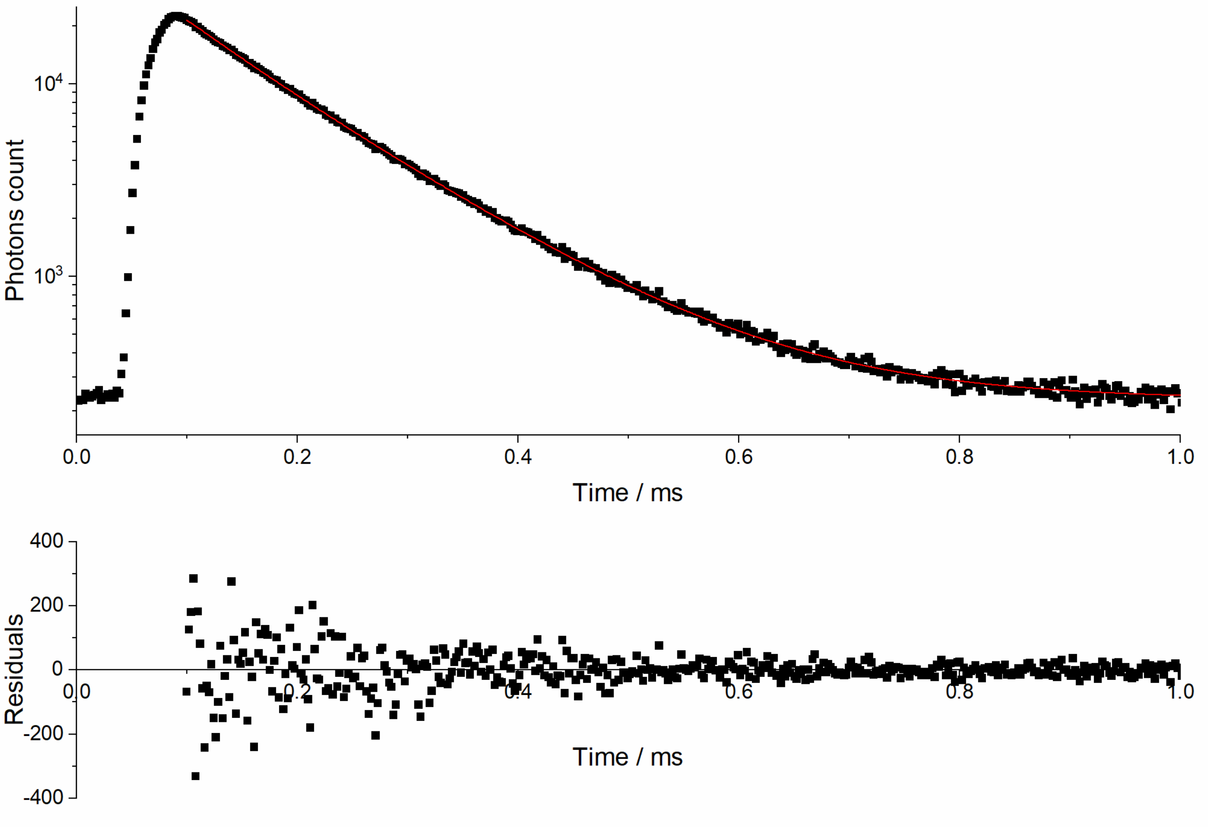

Figure S10. Emission decay profile (black squares, top), corresponding fitted curve (red line) and fit residuals(bottom) for **MoI30@CAP.**

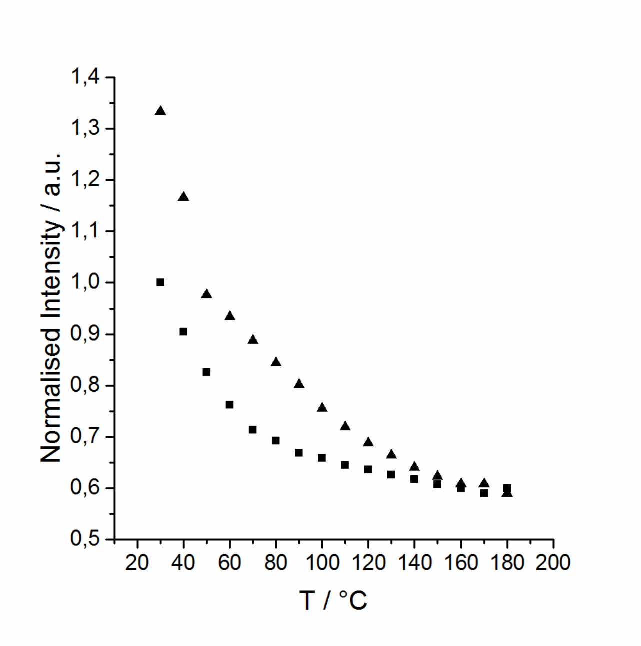

Figure S11. Normalised emission intensity of MoIP1@CAP as function of temperature, (squares) heating cycle, (triangles) cooling cycle.

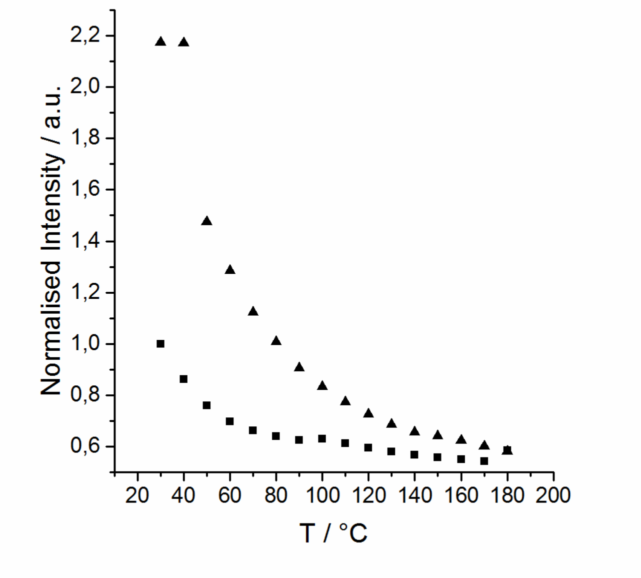

Figure S12. Normalised emission intensity of MoIP10@CAP as function of temperature, (squares) heating cycle, (triangles) cooling cycle.

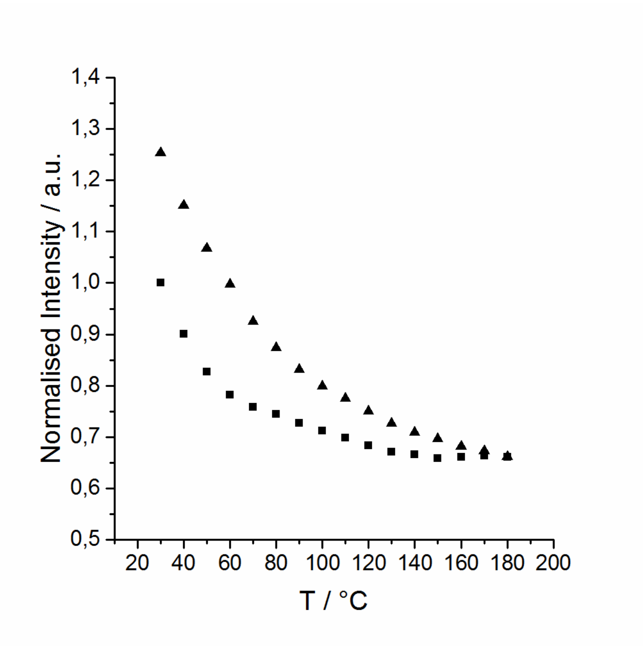

Figure S13. Normalised emission intensity of MoIP20@CAP as function of temperature, (squares) heating cycle, (triangles) cooling cycle.

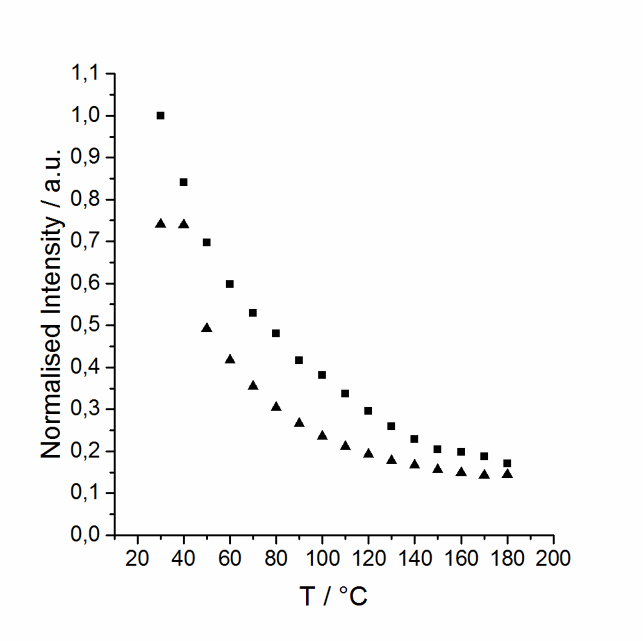

Figure S14. Normalised emission intensity of MoIP30@CAP as function of temperature, (squares) heating cycle, (triangles) cooling cycle.

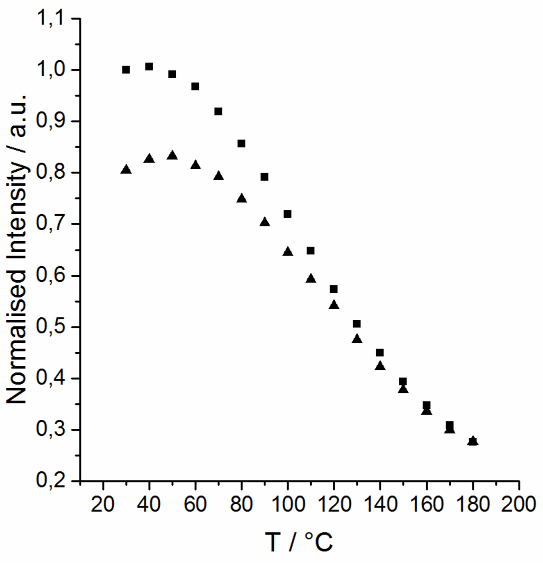

Figure S15. Normalised emission intensity of MoIP as function of temperature, (squares) heating cycle, (triangles) cooling cycle.

1. M. Amela-Cortes, Y. Molard, S. Paofai, A. Desert, J.-L. Duvail, N. G. Naumov, S. Cordier, *Dalton Trans.* 2016, **45**, 237-245.
